# The landscape of neurophysiological outcome measures in ALS interventional trials: A systematic review

**DOI:** 10.1016/j.clinph.2022.02.020

**Published:** 2022-05

**Authors:** N. Ahmed, M.R. Baker, J. Bashford

**Affiliations:** a GKT School of Medical Education, Faculty of Life Sciences and Medicine, King’s College London, UK; b Translational and Clinical Research Institute, Faculty of Medical Sciences, Newcastle University, Newcastle Upon Tyne, UK; c UK Dementia Research Institute, Department of Basic and Clinical Neuroscience, Maurice Wohl Clinical Neuroscience Institute, Institute of Psychiatry, Psychology and Neuroscience, King’s College London, UK

**Keywords:** Neurophysiology, Amyotrophic lateral sclerosis, Electromyography, Motor unit number estimation, Drug trial

## Abstract

**Objective:**

We collated all interventional clinical trials in amyotrophic lateral sclerosis (ALS), which utilised at least one neurophysiological technique as a primary or secondary outcome measure. By identifying the strengths and limitations of these studies, we aim to guide study design in future trials.

**Methods:**

We conducted and reported this systematic review according to the Preferred Reporting Items for Systematic Reviews and Meta-Analyses guidelines. Eight databases were searched from inception. In total, 703 studies were retrieved for screening and eligibility assessment.

**Results:**

Dating back to 1986, 32 eligible interventional clinical trials were identified, recruiting a median of 30 patients per completed trial. The most widely employed neurophysiological techniques were electromyography, motor unit number estimation (including motor unit number index), neurophysiological index and transcranial magnetic stimulation (including resting motor threshold and short-interval intracortical inhibition). Almost 40% of trials reported a positive outcome with respect to at least one neurophysiological measure. The interventions targeted either ion channels, immune mechanisms or neuronal metabolic pathways.

**Conclusions:**

Neurophysiology offers many promising biomarkers that can be utilised as outcome measures in interventional clinical trials in ALS. When selecting the most appropriate technique, key considerations include methodological standardisation, target engagement and logistical burden.

**Significance:**

Future trial design in ALS would benefit from a standardised, updated and easily accessible repository of neurophysiological outcome measures.

## Highlights

• 32 interventional clinical trials in ALS have employed at least one neurophysiological outcome measure.
• The characteristics and experience of ten neurophysiological techniques used in these trials are summarised and evaluated.
• Future trial design would benefit from a more standardised and refined neurophysiological toolkit.

## 1 Introduction

Amyotrophic lateral sclerosis (ALS) is a neurodegenerative disease affecting both upper and lower motor neurons (Kiernan et al., 2011), characterised by loss of glial cells and degeneration of motor neurons in the brain and spinal cord (Wijesekera and Leigh, 2009). ALS is relentlessly progressive with a median survival from symptom onset of 3–5 years (Beghi et al., 2007). There is currently no cure for ALS; the only approved drug in Europe is riluzole (Jaiswal, 2019), while edaravone has also been licensed in Japan and USA.

Despite many clinical trials in ALS, most have yielded negative results. A contributing factor may be the relative insensitivity of trial outcome measures, emphasising the importance of a carefully chosen set of measures relevant to the intervention’s proposed mechanism of action (Beghi et al., 2011). Current outcome measures employed include the revised ALS functional rating scale (ALSFRS-R), forced/slow vital capacity and neurophysiological assessments.

Neurophysiological biomarkers assess motor unit dysfunction, death and/or compensatory adaptations, thereby aiding diagnosis and tracking disease progression in ALS. Their diagnostic importance has been highlighted in both the revised El Escorial criteria (Brooks et al., 2000) and the Gold Coast criteria (Hannaford et al., 2021). Not only can they provide detailed insight into disease mechanisms, but they can also be used as non-invasive outcome measures in therapeutic clinical trials (Vucic and Rutkove, 2018). Moreover, since neurophysiological techniques can detect subclinical disease progression (Neuwirth et al., 2017, Vucic et al., 2008), they have the potential to increase the statistical power of future clinical trials (Sleutjes et al., 2021).

In this systematic review, we provide a comprehensive overview of all ALS clinical interventional trials that have employed neurophysiological outcome measures, assessing their advantages and limitations alongside future recommendations.

## 2 Methods

The reporting of this systematic review is in accordance with the Preferred Reporting Items for Systematic reviews and Meta-Analyses (PRISMA) guidelines (Moher et al., 2009). The protocol for this review has not been published.

### 2.1 Study selection

Studies were included in the review according to the following inclusion criteria: (1) an interventional clinical trial; (2) conducted on patients with ALS; (3) the primary and/or secondary outcome measure(s) included at least one neurophysiological measure; and (4) the study was written in English.

### 2.2 Search strategy

A systematic computerised search was completed by one author (NA) in February 2021 in eight databases: MEDLINE (1946–), PubMed (1972–), Web of Science (1955–), Embase (1947–), PsychInfo, OpenGrey, Cumulative Index to Nursing and Allied Health Literature (CINAHL) and Education Resources Information Centre (ERIC). Searches were tailored to the databases and included key text-word terms, phrases, and medical subject headings (MESH) terms with “and/or” for “amyotrophic lateral sclerosis”, “motor neuron disease”, “clinical trial”, “interventional trial”, “neurophysiological outcome” and related. A full search strategy is presented as a table in Supplementary Table 1. Duplicates amongst the identified studies were removed. The remaining studies were screened by one author (NA), initially based on title and abstract. Of those remaining, the full-text article was assessed, and any queries were resolved by discussion with the senior author (JB). The final list of selected studies was agreed upon by two authors (NA/JB). A repeat search was conducted on 14th October 2021 to include any updates.

### 2.3 Data extraction and quality assessment

Relevant data were extracted from the selected studies in a table with headings as follows: study details (location, sponsors, and type of trial), study design (number of patients and the proposed intervention including mechanism of action), outcome measures, number and duration of follow-ups, results collected, and any strengths/limitations of the neurophysiological measurements used. Additionally, the outcome with respect to the neurophysiological measure has been recorded as positive (where p < 0.05) or negative.

To assess the risk of bias in the selected trials, we used a modified form of the Newcastle-Ottawa Scale (Wells et al., 2000) and the Cochrane Risk of Bias Assessment Tool (Higgins et al., 2011). Both scales assess the risk of bias in three main domains: (1) Selection of the study groups; (2) Comparability between the two groups; and (3) Ascertainment of the outcome and/or the exposure. With the maximum score being 8, studies with a score of ≥7 were considered to have low risk of bias, those with a score of 4–6 were considered to have moderate bias, and scores of <4 were considered to have high risk of bias. We determined adequate follow-up length to be a period of at least 6 months.

## 3 Results

### 3.1 Study selection

**Fig. 1:**
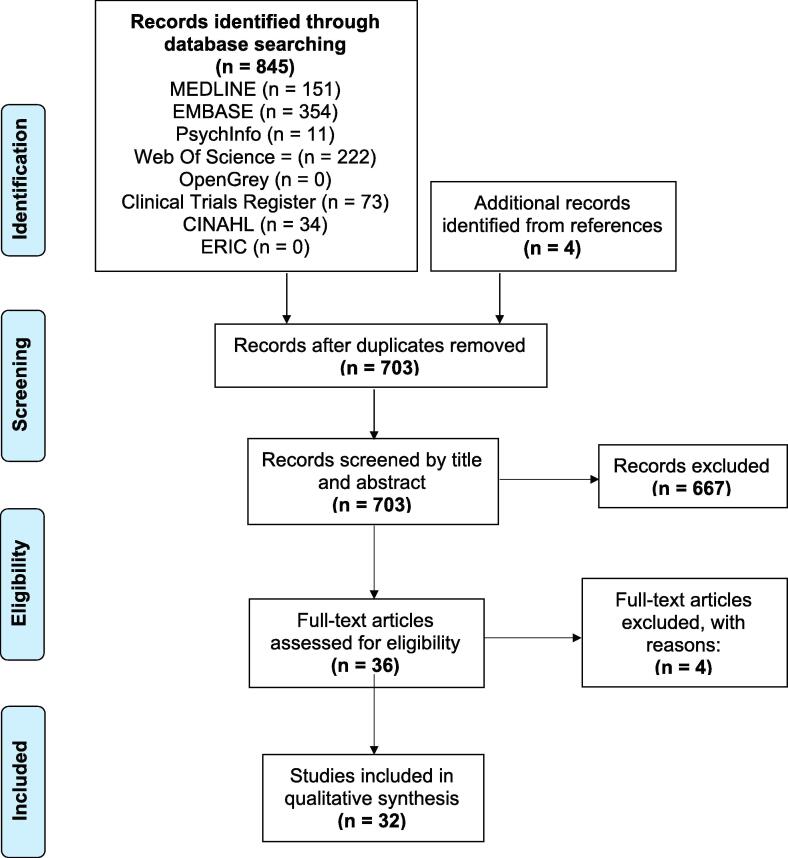
**Flowchart of search strategy and study identification.** Template from: Page MJ, McKenzie JE, Bossuyt PM, Boutron I, Hoffmann TC, Mulrow CD, et al. The PRISMA 2020 statement: an updated guideline for reporting systematic reviews. BMJ 2021;372:n71. https://doi.org/10.1136/bmj.n71. For more information, visit: http://www.prisma-statement.org/ (Page et al., 2021).

A total of 703 studies were obtained from reference and database searches after the removal of duplicates (Fig. 1). The titles and abstracts were screened, and 667 studies were excluded because they did not meet the inclusion criteria. Of the 36 studies remaining, the full text was assessed for eligibility and four were excluded: one did not provide sufficient trial details; one trial had been withdrawn; and the remaining two did not involve neurophysiological outcome measures. Thus, a total of 32 studies were included for qualitative synthesis.

### 3.2 Study and patient characteristics

Eleven of the 32 trials were conducted in Europe (Benussi et al., 2019, de Carvalho et al., 2010, de la Rubia et al., 2019, Desai et al., 1998, Geijo-Barrientos et al., 2020, de la Rubia, 2020, Kuzma-Kozakiewicz et al., 2018, Lingor et al., 2019, Mazzini et al., 2010, Żmijewska and Świątkowska-Flis, 2020, Sommer et al., 1999), nine in North America (Bromberg et al., 2001, Cashman et al., 2008, Cudkowicz et al., 2006, Grifols, 2020, Statland, 2019, Mitsumoto et al., 1986, Shefner et al., 2004, Wainger et al., 2021, Weiss et al., 2021), six in Asia (Li et al., 2017, Nabavi et al., 2019, Nefussy et al., 2010, Petrou et al., 2016, Kuwabara, 2020, Wu, 2020), two in Australia (Park et al., 2015, Vucic et al., 2021a, Vucic et al., 2021b), and four were intercontinental (Bansal et al., 2016, Gold et al., 2019, Kovalchuk et al., 2018, Vucic et al., 2021a, Vucic et al., 2021b). Under half of the trials (41%) were randomised, placebo-controlled and blinded, while the majority of the remainder were reported as either open-label or pilot trials. Sixteen trials (50%) were labelled as either phase 1 (two trials; 6.2%), phase 1/2 (three trials; 9.4%), phase 2 (ten trials; 31.3%), or phase 2/3 (one trial; 3.1%). No trials were labelled as phase 3. Fifteen trials (47%) were conducted over multiple sites. All patients had a diagnosis of definite or probable ALS. The total number of participants from the completed, published trials was 1128, with a median of 30 participants per trial (range: 3–300). Of the completed studies, the mean age varied from 20 to 62.4 years, with 51% of patients being male. The number of estimated patients participating in ongoing studies was 445.

### 3.3 Neurophysiological outcome measures

**Fig. 2:**
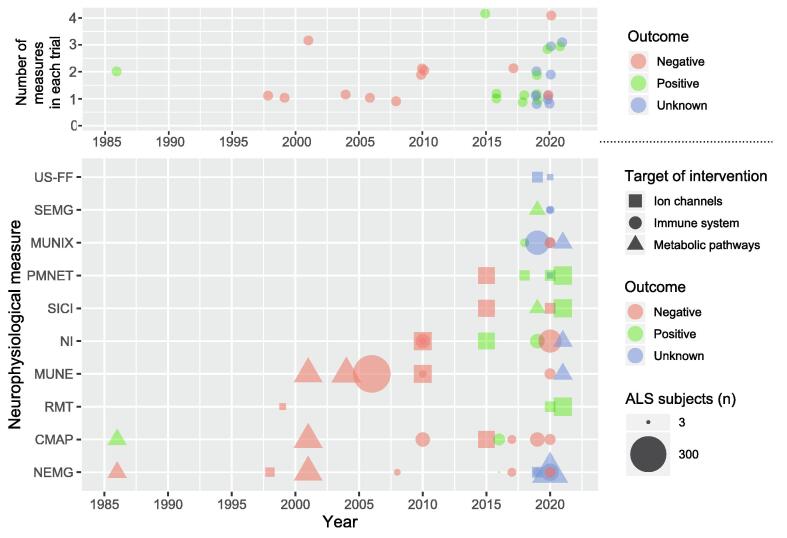
**Timeline of studies.** Since 1986, 32 interventional trials have employed 56 neurophysiological outcome measures in ALS subjects. CMAP, compound muscle action potential; MUNE, motor unit number estimate; MUNIX, motor unit number index; NEMG, needle electromyography; NI, neurophysiological index; PMNET, peripheral motor nerve excitability testing; RMT, resting motor threshold; SEMG, surface electromyography; SICI, short-interval intracortical inhibition; US-FF, ultrasound-fasciculation frequency.

The neurophysiological assessments used were needle electromyography (NEMG; n = 12), compound muscle action potential (CMAP; n = 8), neurophysiological index (NI; n = 7), motor unit number estimation (MUNE; n = 7), motor unit number index (MUNIX; n = 5), peripheral motor nerve excitability testing (PMNET; n = 5), short-interval intracortical inhibition (SICI; n = 4), resting motor threshold (RMT; n = 3), surface electromyography (SEMG; n = 3), and fasciculation frequency detected by ultrasound (n = 2; Table 1*,* Fig. 2*)*. A neurophysiological measure was the primary outcome measure in seven trials (22%), and at least one neurophysiological measure was included as a secondary outcome measure in 28 trials (88%). Fifteen trials (47%) employed a combination of neurophysiological parameters. Final follow-up assessments varied with a median of 4 follow-up assessments per study. The median follow-up time was 1 month with a range of 6 hours – 2 years.

**Table 1:** **The most widely used neurophysiological techniques in ALS interventional trials.** CMAP, compound motor action potential; LMN, lower motor neuron; MUNE, motor unit number estimation; MUNIX, motor unit number index; NEMG, (concentric) needle electromyography; NI, neurophysiological index; PMNET, peripheral motor nerve excitability testing; RMT, resting motor threshold; SICI, short-interval intracortical inhibition.

Outcome measure	Description	Benefits	Drawbacks
CMAP	Supramaximal electrical stimulation of a nerve fibre which produces a response in target muscle being studied. The amplitude corresponds to the number of functional axons.	Quantitative CMAP index is sensitive and can reflect both disease progression alongside detecting subtle changes in motor units (Geijo-Barrientos et al., 2020). Recording can be performed rapidly using non-invasive adhesive pads.	Both nerve stimulation and electrode positioning need to be carefully controlled as to avoid spurious results.
MUNE	Represents an estimation of the number of active motor units in a muscle. The estimate is calculated by dividing the CMAP amplitude to supramaximal nerve stimulation by the mean quantal/increment change in CMAP amplitude (an approximation of the CMAP increase observed by the recruitment of a single motor unit) (de Carvalho et al., 2018).	Provides a quantitative assessment of the number of healthy motor units proving important in measuring disease progression (de Carvalho et al., 2018). Well tolerated by patients as it is non-invasive and can be relatively quick to perform. Can also be easily standardised, with good reproducibility (de Carvalho et al., 2018, Gooch et al., 2014).	Normal findings can often be obtained which reduces its diagnostic sensitivity (de Carvalho et al., 2018). Tolerability and compliance can be affected by the duration of testing.
MUNIX	Assesses the index of motor units upon performance of voluntary muscle contractions at different intensity levels (Vucic and Rutkove, 2018). Using surface interference patterns obtained, along with the CMAP, the index is then calculated using a mathematical model (Nandedkar et al., 2010).	High sensitivity at detecting LMN loss (Vucic and Rutkove, 2018). Well tolerated by patients because of the small number of stimuli needed (Nandedkar et al., 2010).	Requires voluntary muscle activity. Cannot be accurately performed on profoundly weak muscles or in patients where other factors limit voluntary muscle activation (e.g., arthritis) (Vucic and Rutkove, 2018).
NEMG	Provides information on size, morphology and stability of motor unit action potentials and presence/absence of spontaneous motor unit activity (i.e., fasciculation potentials) or spontaneous muscle fibre activity (fibrillation potentials and positive sharp waves).	EMG analysis can be used conclusively alongside clinical parameters in diagnosing ALS (Sengar et al., 2017). Provides a more sensitive marker than clinical parameters with the ability to identify subclinical LMN lesions (Li et al., 2017). Widely available.	Multiple methods exist, and there needs to be an efficient model standardized and used for ALS trials (Sengar et al., 2017). Not well accepted by all patients (Li et al., 2017) - some methods can be quite painful. EMG cannot be used as an acute marker of effects that are expressed continually (Desai et al., 1998).
NI	This index is calculated by utilising three parameters: compound muscle action potential (CMAP), distal motor latency and F wave frequency (de Carvalho et al., 2018, Vucic and Rutkove, 2018).	Irrespective of progression, NI is highly sensitive to LMN loss (de Carvalho et al., 2005). Easily available with a low cost as it does not require special equipment. Can be calculated from standard measurements (de Carvalho et al., 2005). Can be used on patients with spasticity (de Carvalho et al., 2005).	Ceiling/floor effects can be a problem; an index of 0 can be frequently obtained during early follow-up (Nefussy et al., 2010).
PMNET	Reflects membrane permeability, chemical and electrical functions of peripheral nerves. Comprises several measures including strength-duration time constant, threshold electrotonus and current-threshold relationship.	Non-invasive and quick to perform. Correlates well with disease progression with variations mainly due to disease onset (Park et al., 2015).	Using electrodes over the skin means testing must be restricted to areas where the peripheral nerve is near the skin (Kiernan et al., 2005).
RMT	Represents the stimulus intensity required to activate corticospinal connections (Zanette et al., 2002), assessing cortico-motoneuronal excitability.	Well tolerated as procedure was not perceived as painful (Zanette et al., 2002). Cortical excitability differentiates ALS from mimic disorders (e.g., Kennedy’s disease) (Vucic et al., 2011).	Patients with high RMTs will be excluded, reducing potential sample size in trials (Sommer et al., 1999).
SICI	The stimulus intensity required to preserve a target motor evoked potential of 0.2 mV. SICI is assessed using the threshold tracking or paired pulse transcranial magnetic stimulation technique and demonstrates cortical hyperexcitability (Vucic et al., 2013).	Reduction in SICI can precede clinical markers of LMN dysfunction (Vucic and Rutkove, 2018). Robust diagnostic parameter - absent SICI exhibits a sensitivity of 97% (Vucic et al., 2011).	Abnormalities with cortical hyperexcitability have been documented with ALS phenotypes such as flail arm (Vucic and Kiernan, 2007). In patients with high RMTs, testing can be uncomfortable, reducing patient follow up and compliance.

Of the completed trials, 1 out of 7 employing NEMG described an improvement in the outcome measure with the intervention. For CMAP and NI, the positivity rates were 2/8 and 2/6, respectively. Out of the transcranial magnetic stimulation measures, the use of RMT and SICI were associated with positive outcomes in 2/3 and 2/4 trials, respectively. Of the 7 studies using MUNE and/or MUNIX, only 1 reported a positive finding (a MUNIX study). With regards to MUNE, three trials used statistical MUNE, two used the multipoint incremental method, and one used spike-triggering MUNE. Positive effects were seen in 3 out of 4 completed trials reporting PMNET as an outcome measure. Table 2 contains a summary of all trials.

**Table 2:** **List of 32 interventional clinical trials that have employed neurophysiological outcome measures in ALS**. CMAP, compound muscle action potential; FF, fasciculation frequency; MEP, motor evoked potential; MUNE, motor unit number estimate; MUNIX, motor unit number index; NEMG, needle electromyography; NI, neurophysiological index; PMNET, peripheral motor nerve excitability testing; RMT, resting motor threshold; SEMG, surface electromyography; SICI, short-interval intracortical inhibition; US, ultrasound.

Status (Complete [year reported]/ Incomplete)	Randomised? Controlled? Blinded? Phase? Open Label?	Single or multi centre?	Country	Intervention	Mechanism of action	Main neurophysiological measure (1° vs 2°)	Number of ALS patients	Outcome with respect to NP measure (Pos/Neg)
*Interventions acting on neuronal ion channels*								
C (Desai et al., 1998)	R, C, B	S	UK	Riluzole	Inhibits presynaptic release of N-methyl-D-aspartate (NMDA)	NEMG (1°)	15	Negative
C (Sommer et al., 1999)	OL	S	Germany	Riluzole	Inhibits presynaptic release of NMDA	RMT (2°)	8	Negative
C (de Carvalho et al., 2010)	R, C, B, P2/3	S	Portugal	Memantine	NMDA receptor antagonist	MUNE (2°), NI (2°)	63	Negative
C (Park et al., 2015)	R, C, B	M	Australia	Flecainide	Sodium (Na) blocker and membrane stabiliser	CMAP (2°), NI (2°), PMNET (2°), SICI (2°)	54	Positive
C (Kovalchuk et al., 2018)	R, C, B	M	Netherlands, USA	Ezogabine/retigabine	Voltage gated potassium channel (Kv7) agonist	PMNET (1°)	18	Positive
C (Weiss et al., 2021)	R, C, B, P2	M	USA	Mexiletine	Na blocker	RMT (1°), PMNET (2°), SICI (2°)	20	Positive
C (Wainger et al., 2021)	R, C, B, P2	M	USA	Ezogabine/ retigabine	Kv7 agonist	SICI (1°), PMNET (2°), RMT (2°)	65	Positive
I (Statland, 2019)	NR, P2	S	USA	Ranolazine	Na blocker	NEMG-cramp potential duration (2°), US-FF (2°)	20	Not yet known
I (Kuwabara, 2020)	P1/2	S	Japan	Lacosamide	Na blocker	PMNET (2°), SEMG-FF/US-FF (2°)	7	Not yet known

*Interventions targeting the immune system*								
C (Cudkowicz et al., 2006)	R, C, B	M	USA	Celecoxib	Cyclooxygenase (COX) inhibitor	MUNE (2°)	300	Negative
C (Cashman et al., 2008)	Pilot	S	Canada	Granulocyte-colony stimulating factor (G-CSF) mobilized peripheral blood stem cells	Mobilises bone marrow (BM) cells into peripheral blood which can be reinfused to reconstitute normal haematopoiesis	NEMG (1°)	8	Negative
C (Nefussy et al., 2010)	B, Pilot	M	Israel	Recombinant human granulocyte-colony stimulating factor (G-CSF)	Anti-inflammatory and anti-apoptotic effects	CMAP (2°), NI (2°)	39	Negative
C (Mazzini et al., 2010)	OL, P1	M	Italy	Multipotent stem cells	Provide the host tissue with growth factors or modulate the host immune system	MUNE (2°), NI (2°)	10	Negative
C (Bansal et al., 2016)	Case series	S	India	Autologous adult stem cells	Assist neural repair by stimulating synthesis of growth factors which can chaperone/nurse injured tissues and/or by facilitating neurogenesis	NEMG (2°)	3	Positive
C (Petrou et al., 2016)	OL, P1/2 & P2a	S	Israel	Autologous cultured mesenchymal BM stromal cells transplantation	Neurotrophic factor secretions extend motor neuron (MN) survival	CMAP (2°)	26	Positive
C (Li et al., 2017)	OL	S	China	Autologous peripheral blood mononuclear cells	Provide growth factors or modulate host immune system	CMAP (2°), NEMG (2°)	14	Negative
C (Kuzma-Kozakiewicz et al., 2018)	NR, OL	S	Poland	Adipose derived stem cell (ADRC) transplantation	ADRCs improve blood flow, modulate the inflammatory response, and protect tissues from dying.	MUNIX (2°)	14	Negative
C (Nabavi et al., 2019)	OL, P1	M	Iran	Autologous BM derived mesenchymal stromal cells	Trophic factor secretions, ↑interleukin-10 (IL-10) expression and tumour growth factor (TGF)	NEMG (2°)	14	Outcome measure removed
C (Gold et al., 2019)	OL, P2a	M	Australia, UK, USA & Netherlands	Triumeq§	Inhibits human endogenous retroviral activity	NI (2°), CMAP (2 °)	40	Positive
C (Geijo-Barrientos et al., 2020)	R, C, B, P1/2	S	Spain	Bone marrow mononuclear cells	Trophic support and anti-inflammatory actions at axonal/neuromuscular junction (NMJ level).	CMAP (2°), MUNE (2°), MUNIX (2°), NEMG (2°)	22	Negative
C (Vucic et al., 2020)	R, C, B, P2	M	Australia	Dimethyl fumarate (Tecfedira)	Increases ratio of anti-inflammatory regulatory T cells (Tregs)	NI (2°), split hand index (2°)	107	Negative
I (Lingor et al., 2019)	R, C, B, P2a	M	Germany, France & Switzerland	Fasudil	Influences microglial morphology and reduces chemo/cytokine release	MUNIX (2°)	120	Not yet known
I (Grifols, 2020)	OL, P2	S	USA	Plasma exchange (PlEx) with Albutein 5%,	Change the metabolic profile of plasma and cerebrospinal fluid (CSF). Antioxidant effects and detoxifying functions	SEMG-MEP (2°)	12	Not yet known
I (de la Rubia, 2020)	R, B, P2	S	Spain	Resveratrol, curcumin liposomes and dutasteride	Antioxidants	NEMG (1°)	60	Not yet known
I (Żmijewska and Świątkowska-Flis, 2020)	OL, P1/2	M	Poland	Mesenchymal stem cells	Not applicable (N/A)	MUNIX (2°), NEMG (2°)	20	Not yet known

*Interventions affecting neuronal metabolic pathways*								
C (Mitsumoto et al., 1986)	C, B	S	USA	Thyrotropin-releasing hormone	Improve muscle strength + spasticity	CMAP (2°), NEMG (2°)	41	Negative
C (Bromberg et al., 2001)	R, B	M	USA	Branched chain amino acids	N/A	CMAP (2°), MUNE (2°), NEMG (2°)	95	Negative
C (Shefner et al., 2004)	R, B	M	USA	Creatinine	Improves cellular energy buffering + transport systems	MUNE (2°)	104	Negative
C (de la Rubia et al., 2019)	R, C, B	S	Spain	EH301*	Increase nicotinamide adenine dinucleotide (NAD + ) levels and support sirtuin activity	SEMG (2°)	32	Positive
C (Benussi et al., 2019)	R, C, B	S	Italy	Transcranial direct current stimulation	Restoration of intracortical circuits measures	SICI (2°)	30	Positive
I (Wu, 2020)	R, C	S	China	Lipoic acid	Improve motor and respiratory function	NEMG (2°)	150	Not yet known
I (Vucic et al., 2021a, Vucic et al., 2021b)	R, C, B, P2	M	Australia & USA	CNM-Au8	Enhances metabolic capacity of motor neurons	MUNIX (1°), MUNE (2°), NI (2°)	42	Not yet known

* Combination of 1-(β-D-Ribofuranosyl) nicotinamide chloride and 3,5-Dimethoxy-4*′*-hydroxy-trans-stilbene. // § combination of dolutegravir, abacavir and lamivudine.

### 3.4 Study interventions

**Fig. 3:**
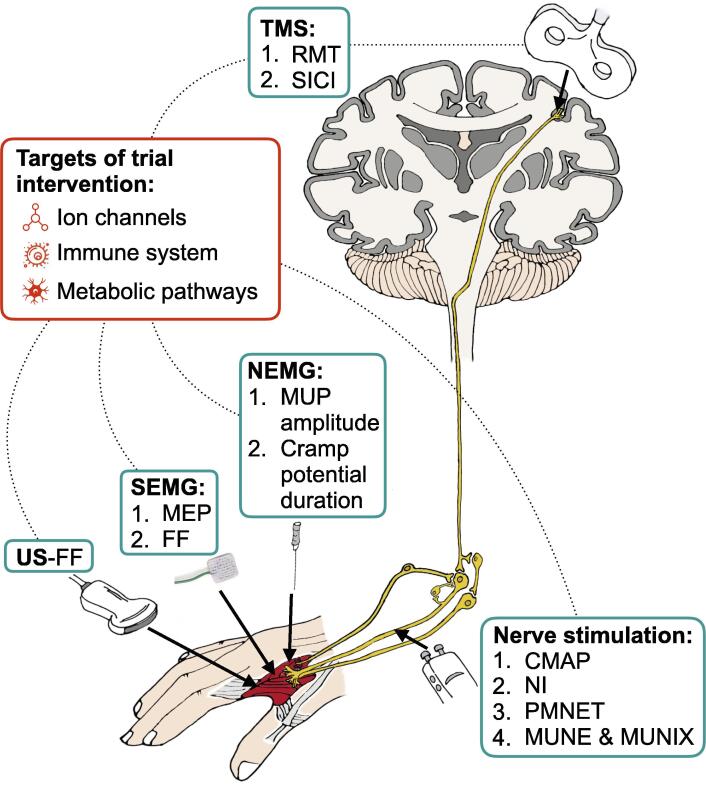
**Summary of neurophysiological measures employed in ALS interventional trials.** CMAP, compound muscle action potential; FF, fasciculation frequency; MEP, motor-evoked potential; MUNE, motor unit number estimate; MUNIX, motor unit number index; MUP, motor unit potential; NEMG, needle electromyography; NI, neurophysiological index; PMNET, peripheral motor nerve excitability testing; RMT, resting motor threshold; SEMG, surface electromyography; SICI, short-interval intracortical inhibition; US, ultrasound.

Almost a third (28%) of the included trials tested an intervention that targeted a neuronal ion channel, mainly sodium-channel blockade, NMDA blockade or potassium-channel agonism (Fig. 3). The other major groups of interventions targeted either the immune system (50%) or neuronal metabolic pathways (22%). Most interventions were oral treatments (52%), while a minority were administered either intravenously (6%), subcutaneously (3%), intrathecally (13%), or using a mixture of methods (26%). Only one trial had a non-pharmacological intervention in the form of transcranial direct current stimulation. The majority of trials (69%) took place during the last ten years. Under half of the completed trials (39%) were positive with respect to at least one neurophysiological outcome measure. A quarter of reported studies remain incomplete.

### 3.5 Study quality

The full risk of bias assessment table is presented in Supplementary Table 2. The study quality ranged from high risk of bias (n = 2) (Desai et al., 1998, Mitsumoto et al., 1986) to a very low risk of bias (n = 2) (Geijo-Barrientos et al., 2020, Park et al., 2015). All included studies accurately assessed the outcome using the neurophysiological measurements detailed above. As defined above, adequate follow up length was determined to be at least 6 months and most studies did not have an adequate follow up, thus reducing the overall quality of the studies. All completed studies highlighted the attrition rate, apart from three studies (Desai et al., 1998, Mitsumoto et al., 1986, Nefussy et al., 2010), which did not describe the loss to follow up. Overall, the studies included in this systematic review had a low risk of bias with 15 of the 24 studies assessed for risk scoring 7 and above. (Bansal et al., 2016, Benussi et al., 2019, Bromberg et al., 2001, Cudkowicz et al., 2006, de Carvalho et al., 2010, de la Rubia et al., 2019, Geijo-Barrientos et al., 2020, Kovalchuk et al., 2018, Li et al., 2017, Nabavi et al., 2019, Park et al., 2015, Shefner et al., 2004, Vucic et al., 2021a, Vucic et al., 2021b, Wainger et al., 2021, Weiss et al., 2021).

## 4 Discussion

Neurophysiological measures have gained increased attention as biomarkers of the neurodegenerative process in ALS (Bashford et al., 2020, de Carvalho et al., 2018, Vucic and Rutkove, 2018). As the natural history of each measure becomes better characterised over time, their implementation as outcome measures in interventional clinical trials has become increasingly robust. In the context of ALS, neurophysiological measures can be categorised into three major groups: (1) Those that pertain to dysfunctional neuronal excitability (e.g., SICI or peripheral excitability testing) (Bashford and Baker, 2020, Vucic et al., 2013); (2) Those that relate directly to neuronal death (e.g., NI or MUNIX) (Neuwirth et al., 2015); and (3) Those that detect a compensatory protective adaptation as a result of neuronal loss (e.g., EMG detection of motor unit reinnervation) (de Carvalho and Swash, 2016). By definition, alterations in neuronal excitability must precede neuronal death in individual neurons, while neuronal death must have already begun within the motor neuron pool to induce compensatory changes in surviving neurons. However, when considering the entire motor neuron pool during a cross-sectional evaluation, all three categories can be captured simultaneously. Certainly, the detection of changes in the living neuron, either in excitability or compensatory adaptations, should be the focus when aiming to retain maximal neuronal function at the earliest stages of disease. By the time significant neuronal death has occurred, it is likely to be too late to halt disease progression and prevent significant disability and death.

We found that interventional clinical trials in ALS have utilised a broad variety of neurophysiological measures. We included US-FF in this review as an imaging modality that detects the mechanical sequela (muscular contraction) of an electrophysiological event (fasciculation potential). The use of other imaging modalities (e.g. MRI) that focus on structural abnormalities were beyond the scope of this review. Although electrical impedance myography has shown potential value as an outcome measure in ALS (Shefner et al., 2018), this systematic review did not identify any interventional trials that have employed this technique.

While standardisation of the included measures was evident for the 47% of trials conducted over multiple sites, we found limited evidence for standardisation between trials. This will undoubtedly have a detrimental effect on the robustness and objectivity of these outcome measures. Key aspects related to standardisation include the choice of muscle(s), sensor type, recording equipment, technical parameters for electrical stimulation (where required), software, analytical processes, and reporting methods (Pugdahl et al., 2010, van den Berg et al., 2019). Ideally, these choices should maximise the cost-effectiveness and global availability of the technique in question, so that attempts by other research groups to replicate a set of results are readily achievable (Neuwirth et al., 2018). The optimal sampling frequency and duration of trials should be decided based on recommendations from previous trials, considering the extra burden placed on patients for more intensive regimes (Rutkove et al., 2020, Shefner et al., 2018, Sleutjes et al., 2021). The relative ability of each outcome measure to detect disease progression in a quantitative and sensitive way should be appreciated, recognising key influences such as test–retest replicability and inter-rater variation (Narayanaswami et al., 2012, Neuwirth et al., 2018, Neuwirth et al., 2016). In this regard, MUNIX and MUNE are amongst the most robust techniques, having been shown to decline by 2.4–9.0% per month in natural history studies (Neuwirth et al., 2015, Neuwirth et al., 2017, Shefner et al., 2011). Appropriate training and assessor validation tests should be clarified and made available by those with sufficient experience of the technique (Fathi et al., 2019). Consensus guidelines detailing the preferred methodology, training requirements, analysis and reporting for each technique would improve the current variations across clinical trials. Such a repository would guide future study design, ensuring that the advantages (e.g., low cost or non-invasiveness) and limitations (e.g., floor effect, reduced patient tolerability) are adequately considered when selecting the best measure for a new trial. It is anticipated that this would eliminate the apparent ‘scatter-gun’ approach revealed in the results of this systematic review.

It was clear from the studies in this systematic review that neurophysiological measures should not be used in isolation. Their combination with clinical, biofluid and imaging biomarkers is vital to ensure a multi-modal output. Direct comparisons between measures from these different categories were limited (9% of trials performed this type of analysis), but it helps to assess how representative the recruited cohort is compared to the wider ALS population (e.g., ALSFRS-R average decline can be compared with other large cohorts) (Chiò et al., 2011, Rooney et al., 2017). It was unsurprising to find that the most commonly reported phase amongst the trials was phase 2 (almost a third), whereby neurophysiological measures (principally as secondary/exploratory outcomes) contributed to decisions over which interventions might benefit from clinical efficacy testing in phase 3. This aligns with recommendations set out in the 2019 revised Airlie House consensus guidelines for the design and implementation of ALS clinical trials, which emphasises that phase 3 outcomes should instead be the remit of well-established survival and functional measures (van den Berg et al., 2019).

It should be highlighted that outcome measures are not the only way neurophysiological techniques can be employed in clinical trials. They can also help to make the recruited cohort more homogenous, reducing the variability and improving the study power (Goyal et al., 2020). This should be chosen based on the presumed mechanism of action of the intervention, ensuring target engagement with the neurophysiological measure used. In some trials, where this approach has been adopted, there was a very clear rationale for the choice of neurophysiological measure. The best example is for the K_v_7 channel agonist, retigabine/ezogabine, which demanded an outcome measure that could detect changes in excitability, more specifically a reduction in pathological hyperexcitability (Kovalchuk et al., 2018). The proposed mechanism of action for some interventions is indirect, for example the COX inhibitor celecoxib, which reduces prostaglandin release, in turn leading to lower glutamate levels (Cudkowicz et al., 2006). Interestingly, for those interventions targeting the immune system, measures of motor neuronal loss (e.g., MUNE, NI) were preferentially chosen, as opposed to measures of neuronal excitability. Another important consideration when matching the best outcome measure with the intervention stems from the neuroanatomical distinction between upper and lower motor neurons. Some neurophysiological measures, such as those obtained by transcranial magnetic stimulation (e.g., SICI, RMT), predominantly detect upper motor neuron disease, while the remainder (e.g., MUNIX, NI) highlight lower motor neuron degeneration.

## 5 Conclusion

It is widely agreed that neurophysiological outcome measures can play a significant role in the assessment of novel drug effectiveness in ALS. In this review, we have highlighted 32 clinical trials that have put a wide variety of these tools to the test, involving interventions with disparate mechanisms of action. While NEMG demonstrated relatively poor utility in this context, newer techniques such as MUNIX and SICI showed the greatest promise. What is evident from this analysis is that greater standardisation between trials is required, especially as trials begin to include greater numbers of patients, spanning multiple international sites. This would be particularly important for emerging neurophysiological techniques to ensure there is a solid knowledge and experience base for the future. In selecting the most appropriate outcome measure(s), the relative strengths and pitfalls of each should be considered, whilst ensuring target engagement with the intervention’s mechanism of action. In this regard, we hope this analysis can serve as a guide for future trial design in this field.

## Declaration of Competing Interest

The authors declare that they have no known competing financial interests or personal relationships that could have appeared to influence the work reported in this paper.
