# Supplementary material for: The landscape of neurophysiological outcome measures in ALS interventional trials: A systematic review
Source: Clin Neurophysiol. 2022 May;137:132–41. doi: 10.1016/j.clinph.2022.02.020 (PMC10166714; doi:10.1016/j.clinph.2022.02.020)
Supplement: Supplementary data 1 [file mmc1.docx]

| **Objective** | **Key words** | **Synonyms** |
| --- | --- | --- |
| Population | ALS patients | Amyotrophic lateral sclerosis ***OR*** ALS  ***OR*** Motor neuron disease ***OR*** MND  **OR** Progressive muscular atrophy ***OR*** PMA  ***OR*** Primary lateral sclerosis ***OR*** PLS  ***OR*** Flail limb ***OR*** flail arm ***OR*** flail leg variants  ***OR*** Lou Gehrig’s disease  ***AND*** |
| Intervention | Clinical trial | Drug trial ***OR*** Clinical trial ***OR*** Interventional trial  ***AND*** |
| Outcome measure | Neurophysiological   - MUNIX - SICI | Neurophysiological outcome  ***OR*** MUNIX ***OR*** Motor unit number index  ***OR*** SICI ***OR*** Short-interval intra-cortical inhibition ***OR*** Short interval intracortical inhibition  ***OR*** Neurophysiological index ***OR*** NI  ***OR*** Electromyography ***OR*** EMG  ***OR*** Transcranial magnetic stimulation ***OR*** TMS  ***OR*** Resting motor threshold ***OR*** RMT  ***OR*** Axonal excitability  ***OR*** Peripheral excitability studies  ***OR*** Motor unit number estimate ***OR*** MUNE  ***OR*** Electrical impedance myography ***OR*** EIM |

**Supplementary Table 1.** Detailed search strategy used in this systematic review.

Inclusion criteria:

- Interventional clinical trial (preferably randomised, placebo-controlled)
- Involves ALS patients
- A neurophysiological measure included as either primary or secondary outcome (mainly MUNIX/SICI)

| Study | Selection | | | Comparability | Outcome | | | Overall score /8 |
| --- | --- | --- | --- | --- | --- | --- | --- | --- |
|  | Adequate sample definition | Representativeness of sample | Selection of controls |  | Assessment of outcome | Adequate follow up length | Adequacy of follow up |  |
| [Weiss (2020)](https://onlinelibrary.wiley.com/doi/10.1002/mus.27146) | * | * | * | ** | * |  | * | 7 |
| [Wainger (2021)](https://clinicaltrials.gov/ct2/show/NCT02450552?term=NCT02450552) | * | * | * | ** | * | * |  | 7 |
| [Geijo-Barrientos (2020)](https://www.frontiersin.org/articles/10.3389/fnins.2020.00195/full) | * | * | * | ** | * | * | * | 8 |
| [Lingor (2019)](https://www.frontiersin.org/articles/10.3389/fneur.2019.00293/full) |  |  |  |  |  |  |  | N/A |
| [Rubia (2019)](https://www.tandfonline.com/doi/full/10.1080/21678421.2018.1536152?scroll=top&needAccess=true) | * |  | * | ** | * | * | * | 7 |
| [Benussi (2019)](https://www.sciencedirect.com/science/article/pii/S1935861X19302621) | * |  | * | ** | * | * | * | 7 |
| [Kovalchuk (2018)](https://ascpt.onlinelibrary.wiley.com/doi/full/10.1002/cpt.1096?saml_referrer=) | * | * | * | ** | * |  | * | 7 |
| [Bansal (2016)](https://search.proquest.com/openview/e4666b55a47a033cee874d682278aaf1/1?pq-origsite=gscholar&cbl=2034826) | * | * |  | ** | * | * | * | 7 |
| [Park (2015)](https://www.sciencedirect.com/science/article/pii/S2352396415302061#!) | * | * | * | ** | * |  | * | 7 |
| [Carvalho (2010)](https://www.tandfonline.com/doi/full/10.3109/17482968.2010.498521) | * | * | * | ** | * | * | * | 8 |
| [Nefussy (2010)](https://www.researchgate.net/profile/Vivian-Drory/publication/24434109_Recombinant_human_granulocyte-colony_stimulating_factor_administration_for_treating_amyotrophic_lateral_sclerosis_A_pilot_study/links/0deec532dfb3900c80000000/Recombinant-human-granulocyte-colony-stimulating-factor-administration-for-treating-amyotrophic-lateral-sclerosis-A-pilot-study.pdf) | * |  | * | ** | * | * |  | 6 |
| [Desai (1998)](https://www.sciencedirect.com/science/article/pii/S0022510X98002019) |  |  | * |  | * |  |  | 2 |
| [Sommer (1999)](https://link.springer.com/article/10.1007/BF03161086) | * |  |  | ** | * |  | * | 5 |
| [Mitsumoto (1986)](https://ovidsp.dc1.ovid.com/ovid-b/ovidweb.cgi?WebLinkFrameset=1&S=OMBFFPEDDMACPLJMKPPJJHKIHPNCAA00&returnUrl=ovidweb.cgi%3fMain%2bSearch%2bPage%3d1%26S%3dOMBFFPEDDMACPLJMKPPJJHKIHPNCAA00&fromjumpstart=1&directlink=https%3a%2f%2fovidsp.dc1.ovid.com%2fovftpdfs%2fFPACKPKIJHJMDM00%2ffs047%2fovft%2flive%2fgv024%2f00006114%2f00006114-198602000-00003.pdf&filename=Amyotrophic+lateral+sclerosis%3a+Effects+of+acute+intravenous+and+chronic+subcutaneous+administration+of+thyrotropin-releasing+hormone+in+controlled+trials.&navigation_links=NavLinks.S.sh.22.1&link_from=S.sh.22%7c1&pdf_key=FPACKPKIJHJMDM00&pdf_index=/fs047/ovft/live/gv024/00006114/00006114-198602000-00003&D=ovft&link_set=S.sh.22\|1\|sl_10\|resultSet\|S.sh.22.23\|0) | * |  |  | * | * |  |  | 3 |
| [Vucic (2021)](https://www.ncbi.nlm.nih.gov/pmc/articles/PMC7802642/) |  | - | - | - | - | - | - | N/A |
| [Li (2017)](https://www.ncbi.nlm.nih.gov/pmc/articles/PMC5399730/) | * | * |  | ** | * | * | * | 7 |
| [Cashman (2008)](https://onlinelibrary.wiley.com/doi/full/10.1002/mus.20951?saml_referrer) | * |  |  |  | * | * | * | 4 |
| [Gold (2019)](https://www.tandfonline.com/doi/pdf/10.1080/21678421.2019.1632899) | * |  |  | ** | * |  | * | 5 |
| [Kuzma-Kozakiewicz (2018)](https://www.hindawi.com/journals/sci/2018/4392017/) | * | * |  | * | * | * | * | 6 |
| [Cudkowicz (2006)](https://onlinelibrary.wiley.com/doi/full/10.1002/ana.20903?saml_referrer) | * |  | * | ** | * | * | * | 7 |
| [Bromberg (2001)](https://www.sciencedirect.com/science/article/abs/pii/S0022510X00004895) | * | * | * | * | * | * | * | 7 |
| [Nabavi (2019)](https://www.ncbi.nlm.nih.gov/pmc/articles/PMC6099146/) | * | * |  | ** | * | * | * | 7 |
| [Zhi-Ying Wu (2020)](https://clinicaltrials.gov/ct2/show/NCT04518540#wrapper) | - | - | - | - | - | - | - | N/A |
| [José (2020)](https://clinicaltrials.gov/ct2/show/NCT04654689#wrapper) | - | - | - | - | - | - | - | N/A |
| [Flex pharma (2018)](https://clinicaltrials.gov/ct2/show/NCT03338114#moreinfo) | N/A | N/A | N/A | N/A | N/A | N/A | N/A | N/A |
| [Statland (2020)](https://clinicaltrials.gov/ct2/show/NCT03472950#wrapper) | - | - | - | - | - | - | - | N/A |
| [Grifols Therapeutics (2020)](https://clinicaltrials.gov/ct2/show/study/NCT02872142#wrapper) | - | - | - | - | - | - | - | N/A |
| [Beata Świątkowska-Flis (2020)](https://clinicaltrials.gov/ct2/show/NCT04651855) | - | - | - | - | - | - | - | N/A |
| [Petrou (2016)](https://jamanetwork.com/journals/jamaneurology/fullarticle/2480881) | * | * |  | * | * | * | * | 6 |
| [Satoshi Kuwabara (2020)](https://clinicaltrials.gov/ct2/show/NCT03186040#wrapper) | - | - | - | - | - | - | - | N/A |
| [Shefner (2004)](https://oce.ovid.com/article/00006114-200411090-00022/HTML) | * | * | * | ** | * |  | * | 7 |
| [Vucic (2020)](https://www.ncbi.nlm.nih.gov/pmc/articles/PMC7015658/) | * | * | * | ** | * |  | * | 7 |
| [Mazzini (2009)](https://www.sciencedirect.com/science/article/pii/S0014488609003173) | * | * |  | * | * | * | * | 6 |

**Supplementary Table 2.** Detailed risk of bias assessment for each study identified.
